# Mesoporous Bioactive Glasses: A Review on Structure-Directing-Based Synthesis, Characterization, and Biomedical Applications

**DOI:** 10.3390/ma19050876

**Published:** 2026-02-26

**Authors:** Adriana Vulpoi, Ioan Botiz

**Affiliations:** 1 Nanostructured Materials and Bio-Nano-Interfaces Center, Interdisciplinary Research Institute on Bio-Nano-Sciences, Babeș-Bolyai University, 400271 Cluj-Napoca, Romania; adriana.vulpoi@ubbcluj.ro; 2 Contrast Agents and Specific Therapeutics Center, INSPIRE Platform, Interdisciplinary Research Institute on Bio-Nano-Sciences, Babes-Bolyai University, 400271 Cluj-Napoca, Romania; 3 Department of Physics of Condensed Matter and Advanced Technologies, Faculty of Physics, Babeș-Bolyai University, 400084 Cluj-Napoca, Romania

**Keywords:** mesoporous bioactive glass, structure directing agent, sol–gel evaporation-induced self-assembly, biomedical applications

## Abstract

Mesoporous bioactive glasses (MBGs) represent a significant advancement in bioactive glass technology, combining the well-established osteoconductive and osteoinductive properties of traditional bioactive glasses with the structural precision provided by highly ordered mesoporosity. Their characteristic architecture, defined by uniform pores typically ranging from a few to several tens of nanometers and exceptionally high surface areas reaching several hundred m^2^/g, enables enhanced drug-loading capacity, controlled therapeutic ion release, and accelerated tissue regeneration. In this work, we emphasize how the synthesis of these materials is predominantly governed by structure-directing agents, which critically influence the pore size, mesophase ordering, surface area, and structural stability. Additionally, we discuss how compositional tailoring, particularly through therapeutic ion doping with elements such as Sr, Cu, Zn, or B, can impart osteogenic, angiogenic, antibacterial, or antioxidant functionalities. Moreover, we illustrate how these functionalities can be further expanded and enhanced by employing a comprehensive suite of characterization tools to establish robust correlations between synthesis parameters, mesostructural features, and biological performance. Improving the above functionalities enables the MBGs to exhibit exceptional versatility across biomedical applications, notably in bone tissue engineering (as hierarchical or composite scaffolds), controlled drug delivery (anticancer, antibiotic, and anti-inflammatory agents), wound healing, dental therapy, and bioactive implant coatings. Finally, we acknowledge that despite their broad potential, several associated challenges remain, including the synthesis scalability, batch-to-batch reproducibility, mechanical fragility of pure MBGs, and the complexity of predicting in vivo degradation and ion-release behaviors. We believe that emerging research directions, including eco-friendly synthesis routes, stimuli-responsive smart MBGs, multifunctional theranostic platforms, and patient-specific additive manufacturing, are poised to overcome current limitations and drive the next generation of MBG-based biomedical technologies.

## 1. Introduction

Mesoporous bioactive glass (MBG) represents a unique class of silica-based biomaterials that combine the intrinsic bioactivity of conventional bioactive glasses with the high surface area, uniform porosity, and structural tunability characteristic of mesoporous materials [1,2]. Their typical pore sizes—ranging from a few to several tens of nanometers—facilitate strong bonding to bone tissue and promote the formation of new mineralized matrix [3,4,5]. While modern MBG technology is grounded in mesostructure engineering, its conceptual foundation can be traced back to early bioactive glass research. The development of bioactive glasses began in 1969 with Hench’s creation of 45S5 Bioglass^®^, a composition capable of bonding to bone through the formation of a hydroxyapatite (HA) layer in physiological fluids [6]. Beyond the classical 45S5 composition, newer silicate-based bioactive glasses such as 55S, as well as bioactive borate glasses (e.g., 13-93B3), have been developed to achieve faster degradation rates, enhanced ion release, and improved processing versatility for specific biomedical applications [7,8,9]. Conventional bioactive glasses pioneered clinical applications in bone repair but were limited by low surface area and lack of controlled porosity, which restricted drug loading, ion exchange, and biological performance [6,10,11].

MBGs typically follow the SiO_2_–CaO–P_2_O_5_ system [6,7], where ordered mesopores are generated through surfactant-templated synthesis strategies that promote nanostructured organization [2,12], with novel strategies being continuously developed. These architectures often achieve surface areas ranging from several hundred to over 1000 m^2^/g [6,13], providing extensive space for drug incorporation and controlled release. The resulting interconnected porosity enhances mass transport, accelerates mineralization, and supports finely tuned therapeutic ion delivery. Defined by IUPAC as materials with pore diameters between 2 and 50 nm, MBGs frequently display uniform channels in the 3–10 nm range and surface areas of 200–700 m^2^/g [2,14]. The key to generating these mesostructures lies in the strategic use of surfactants and polymers as structure-directing agents (SDAs). Surfactants such as nonionic triblock copolymers (TBCs; e.g., P123, F127, and F68) or cationic surfactants (e.g., CTAB) self-assemble into micelles that act as templates around which silicate precursors condense, yielding ordered porosity upon removal of the organic template [2,12]. Adjusting synthesis conditions enables tuning of Si, Ca, and P content [15,16,17,18,19], as well as incorporation of dopants such as Sr, Zn, or Cu to induce osteogenic, angiogenic, antibacterial, or anti-inflammatory responses [20,21,22,23]. Such templating strategies underpin sol–gel and evaporation-induced self-assembly (EISA) routes widely used to achieve precise mesostructural control in MBGs [2,14,16].

MBGs exhibit excellent biocompatibility, supporting cell adhesion, proliferation, and hydroxyapatite formation when immersed in physiological conditions [24,25,26,27,28]. Their surface reactivity and ion-release capabilities enable effective interaction with biological environments, promoting tissue regeneration. These combined properties have significantly expanded the functional applications of MBGs beyond those of traditional bioactive glasses. Currently, MBGs are utilized in regenerative medicine [10,29]; bone regeneration as scaffolds and coatings [2,15,30], tissue engineering as 3D supports [6,27,31,32], wound healing [33,34,35,36], gene delivery [37], controlled drug delivery systems [14,38,39,40], antimicrobial applications [10,41], and bioimaging [41]. Their incorporation into composite scaffolds and implant coatings further enhances osteoconductivity, antibacterial performance, and mineralization capacity [6,10].

Polymers play a central role in advancing MBG structuring strategies, as they are versatile materials capable of generating ordered micro- and nanostructures through both top–down and bottom–up approaches [42,43,44,45,46]. Their applications span medicine [47,48], bio-inspired materials [49,50], optoelectronics [51], and beyond [52,53,54]. In MBG synthesis, polymers serve as templates, stabilizers, functional modifiers, and matrix materials, enabling precise control over pore geometry, surface chemistry, degradation behavior, and mechanical properties [55,56,57,58,59,60,61,62,63,64,65]. Polymer–MBG hybrids expand application possibilities by combining structural flexibility with bioactive ion release. These converging advances in templating systems, polymer-assisted assembly, and compositional control form the foundation of modern MBG design.

In this work, we present a comprehensive review of synthesis mechanisms, main SDAs, compositional effects, characterization techniques, biomedical applications, and future challenges in MBG research.

In mesoporous bioactive glasses, synthesis parameters and base composition jointly determine mesostructural features such as pore size, ordering, and surface area. These structural characteristics directly influence degradation behavior and ion-release kinetics, which in turn govern biological responses including hydroxyapatite formation, cell interaction, and tissue regeneration [17,66]. Establishing clear cause-and-effect relationships linking synthesis, structure, and biological performance is therefore essential for the rational design of MBGs tailored to specific biomedical applications.

## 2. Synthesis Mechanisms and Methods Used to Obtain MBGs

**Figure 1:**
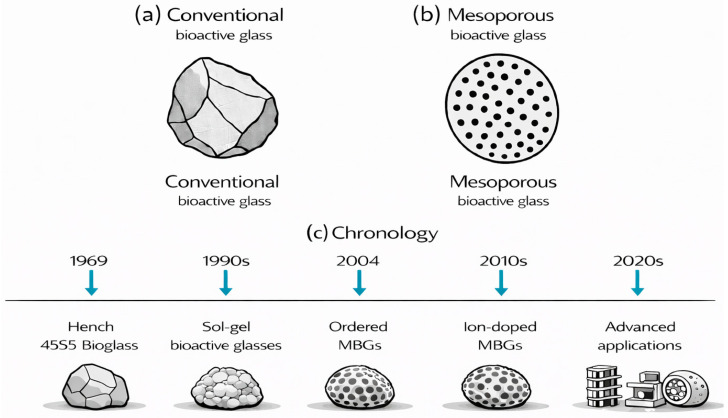
(**a**,**b**) Schematic comparison of conventional (**a**) and mesoporous (**b**) bioactive glass. (**c**) The historical context and evolution of MBGs.

**Figure 2:**
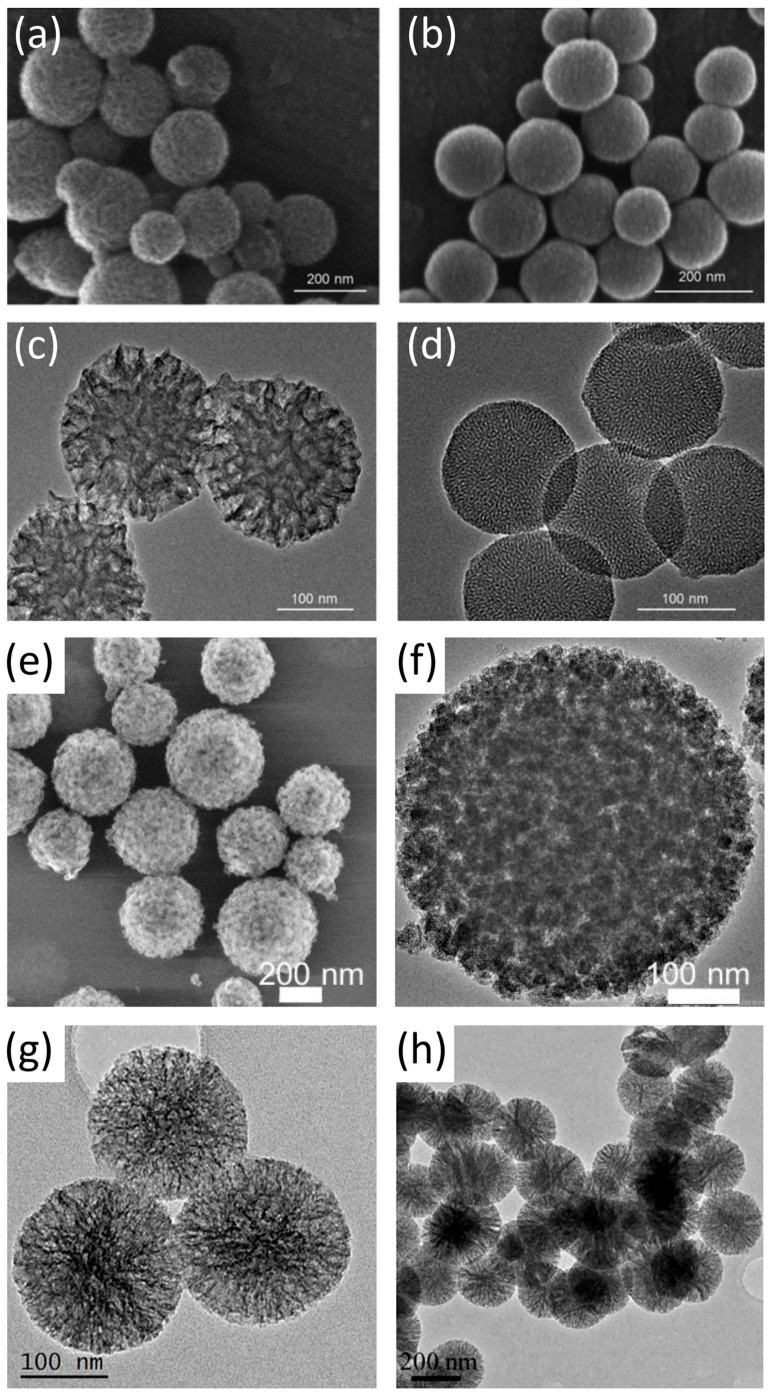
(**a**,**b**) SEM and (**c**,**d**) TEM images emphasizing mesoporous bioactive glass nanoparticles with large (**a**,**c**) and small (**b**,**d**) pores. (**e**,**f**) TEM micrographs depicting calcined samples of hierarchically porous bioactive glass. (**g**,**h**) TEM images emphasizing alginate-modified MBG. Reproduced with permission from ref. [68] (**a**–**d**) [Published by The Royal Society of Chemistry], ref. [69] (**e**,**f**) [Copyright (2023) by the authors. Licensee MDPI, Basel, Switzerland] and ref. [70] (**g**,**h**) [Copyright (2022), Yao, Luo, Deng, Li and Wei].

Compared to conventional bioactive glasses, which exhibit low surface area and lack significant porosity, factors that severely limit drug loading capacity and biological performance, MBGs consist of ordered mesopores created through surfactant-templated methods. These methods produce nanostructured architectures with exceptionally large surface areas that can be loaded with substantial amounts of drugs (Figure 1a,b). The synthesis and development of MBGs, from the late 1960s to the present, are illustrated in Figure 1c. More precisely, various MBGs (Figure 2) can be synthesized through several polymer-assisted routes, in which surfactant or polymer assemblies guide the formation of ordered pore networks [14,21,25,27,67,68,69,70]. The most widely used approaches include the surfactant-templated sol–gel process, EISA [5,40,61], the modified Stöber method [71,72,73,74,75], microemulsion-assisted sol–gel synthesis [76,77,78,79], and polymer-composite processing [15,35,55,57]. Across these strategies, amphiphilic molecules regulate micelle formation, pore geometry, and particle morphology, enabling reproducible mesostructures suitable for biomedical applications. The following subsections outline these mechanisms, focusing on polymer-directed organization and key structural parameters.

### 2.1. Surfactant-Templated Sol–Gel and Evaporation-Induced Self-Assembly (EISA)

**Figure 3:**
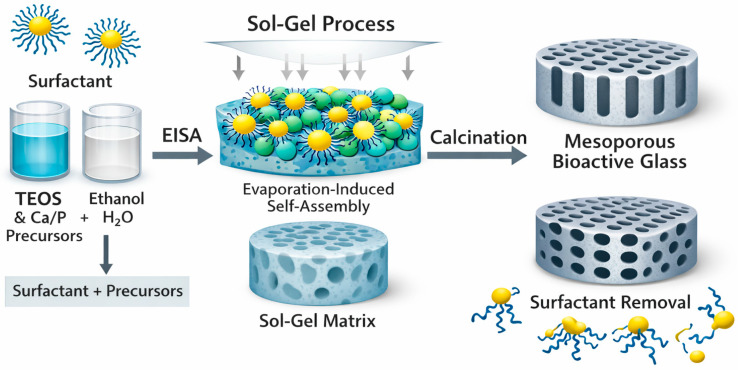
Typical formation of a MBG by EISA. Surfactant-directed self-assembly during the sol–gel process generates an ordered hybrid matrix, which after calcination yields MBG with uniform mesoporous channels.

In the surfactant-templated sol–gel method, amphiphilic molecules such as CTAB, P123, and F127 self-assemble into micelles that serve as structure-directing templates for mesopore formation [6]. Hydrolyzed silicate species derived from TEOS condense around these micelles, generating ordered mesostructures once the organic template is removed [14,21,80]. During the sol–gel transition, the organization of the micellar phase determines the final pore symmetry and surface area of the material. EISA, schematically illustrated in Figure 3, provides further control by promoting micelle ordering through gradual solvent evaporation, which increases surfactant concentration and facilitates packing into periodic mesophases such as hexagonal or cubic lattices [1,5,61]. This approach is particularly effective for thin films and coatings, where the evaporation rate directly influences mesopore alignment and thickness. Within this framework, TEOS hydrolysis typically occurs in acidic ethanol-based solutions, incorporating Ca and P precursors in controlled stoichiometric ratios [14,16]. The subsequent condensation step is strongly influenced by pH, which is usually maintained between 1 and 2 to accelerate Si–OH formation and promote ordered network assembly [16,19,80]. Template removal is achieved by calcination, typically performed at 600–700 °C with slow heating rates to preserve the mesostructure, preventing pore collapse or premature crystallization [59,81].

### 2.2. Representative P123-Templated MBG Protocol

Pluronic P123 is commonly used as a SDA due to its amphiphilic block copolymer configuration, which forms well-defined micelles capable of producing uniform mesopores [14,20,31]. Its hydrophilic–hydrophobic architecture allows precise tuning of pore size, wall thickness, and particle morphology. A typical synthesis involves dissolving P123 in ethanol, combining it with TEOS, and allowing hydrolysis under acidic conditions, followed by the incorporation of Ca(NO_3_)_2_·4H_2_O and TEP at defined molar ratios [14,16]. Aging promotes micelle ordering, before the thermal treatment at 600–700 °C removes the template and stabilizes the mesoporous network. This procedure consistently yields MBGs with highly ordered pores and compositions suitable for drug release and osteogenic applications.

### 2.3. Critical Synthesis Parameters

Several key parameters—including surfactant concentration, pH, solvent polarity, and aging temperature—govern micelle formation and ultimately control pore size, ordering, and structural fidelity [14,82,83] (Table 1). Fine-tuning these parameters allows for the optimization of surface area, pore volume, and ion-release profiles. The TEOS-to-surfactant ratio defines micelle packing and pore symmetry, with P123-based syntheses frequently using ratios of 10–40 to achieve ordered mesophases [14]. Maintaining an acidic pH favors controlled hydrolysis, whereas borate-based MBGs often require milder catalysts, such as citric acid, to avoid rapid, uncontrolled reactions [31,36]. Aging typically occurs between 60 and 80 °C, promoting micelle assembly and silicate condensation. However, borate systems can consolidate more rapidly, often within 12 to 24 h [36]. Calcination at 600 to 700 °C ensures template removal and network stabilization, whereas borate and phosphate systems generally require lower temperatures of 500 to 600 °C to prevent volatilization or crystallization [59,81].

### 2.4. Modified Sol–Gel Methods

**Figure 4:**
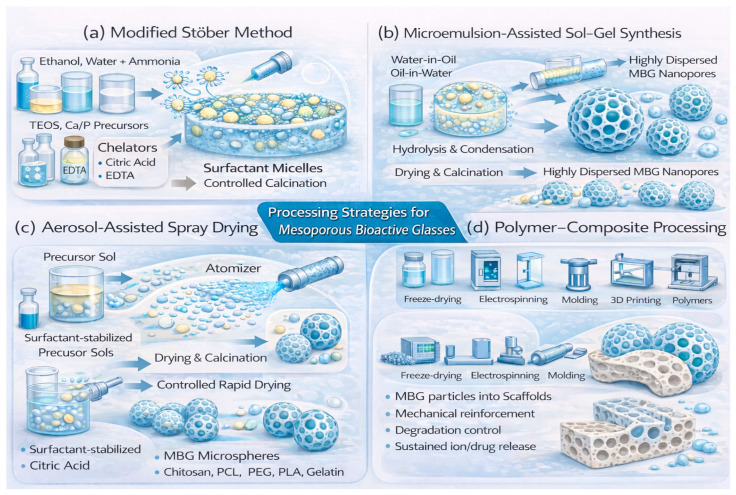
Processing strategies for MBGs. (**a**) Modified Stöber (surfactant-assisted sol–gel) synthesis produces spherical, monodisperse mesoporous nanoparticles via micelle-directed TEOS hydrolysis and condensation, with Ca/P incorporation, optional therapeutic dopants, and controlled calcination. (**b**) Microemulsion-assisted sol–gel synthesis uses surfactant-stabilized droplets as nanoreactors to yield highly dispersed MBG nanoparticles with tunable mesoporosity. (**c**) Aerosol-assisted spray drying generates mesoporous MBG microspheres (∼1–10 µm) through rapid solvent evaporation and simultaneous micelle ordering. (**d**) Polymer–composite processing integrates MBGs into polymer matrices to form hybrid scaffolds with enhanced mechanical properties and controlled ion or drug release.

Some typical sol–gel methods used to generate various MBGs are schematically depicted in Figure 4. The modified Stöber method adapts the classical Stöber silica synthesis by introducing surfactants into ethanol–water–ammonia media, where micelles direct the hydrolysis and condensation of TEOS into spherical mesoporous nanoparticles [71,72,73,74,75]. This approach produces monodisperse particles with well-defined mesopores, making them ideal for drug delivery and injectable systems. The hydrolysis of TEOS is accompanied by the incorporation of Ca and P sources, which confer bioactivity and enable the material to form hydroxyapatite upon contact with physiological fluids. Additional dopants such as Zn^2+^, Cu^2+^, or Sr^2+^ can be introduced to impart therapeutic functionalities, including antibacterial and angiogenic effects [36,56]. For borate and phosphate MBGs, precursor reactivity requires more controlled conditions, including lower calcination temperatures—typically 550–600 °C for borates and 400–500 °C for phosphate-rich systems [39,61,84]. Chelating additives such as citric acid or ethylenediaminetetraacetic acid (EDTA) stabilize hydrolysis and prevent premature network collapse, enabling mesophase formation even at high phosphate contents.

### 2.5. Microemulsion-Assisted Sol–Gel Synthesis

Microemulsion systems—comprising water-in-oil or oil-in-water droplets stabilized by surfactants—serve as nanoreactors where TEOS hydrolysis and condensation take place, producing monodisperse MBG nanoparticles [35,82,85,86]. This confined-space chemistry yields uniform particle size, high dispersion, and tunable porosity. These droplets incorporate silica precursors along with Ca and P sources, generating spherical MBG nanoparticles suitable for injectable formulations and drug-release systems. The ratios of surfactants and co-surfactants strongly influence particle diameter, pore characteristics, and dispersibility.

### 2.6. Aerosol-Assisted Spray Drying

In aerosol-assisted spray drying, precursor sols containing surfactants are atomized into fine droplets. As the solvent rapidly evaporates, micelle ordering and silica condensation occur simultaneously, producing mesoporous microspheres [55]. This method offers excellent scalability and precise control over particle size. The resulting MBG microspheres typically exhibit mesoporous shells and narrow size distributions, generally ranging from 1 to 10 µm, making them well-suited for large-scale production [16].

### 2.7. Polymer–Composite Processing

**Figure 5:**
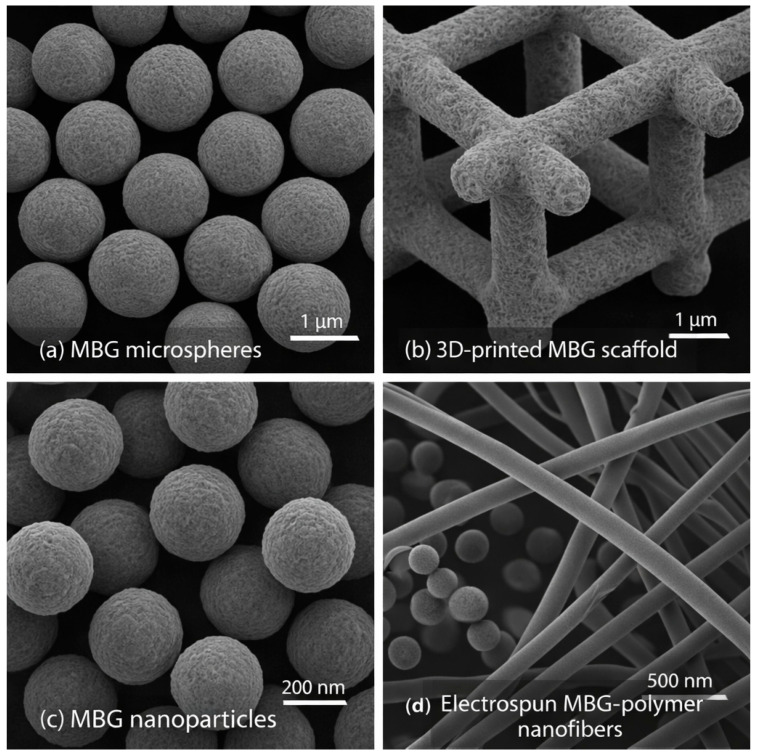
Schematic, the literature-inspired images representing typical morphologies of MBG–based structures generated solely for illustrative purposes. (**a**) MBG microspheres with uniform spherical morphology. (**b**) 3D-printed MBG scaffold showing an interconnected macroporous architecture. (**c**) MBG nanoparticles with monodisperse nanoscale dimensions. (**d**) Electrospun MBG–polymer composite nanofibers forming a fibrous network suitable for tissue engineering applications.

Polymer–composite methods incorporate MBG powders or nanoparticles into natural or synthetic polymer matrices such as chitosan, PCL, PEG, PLA, or gelatin, resulting in hybrid scaffolds with enhanced mechanical properties [15,35,55,57]. These composites combine the structural flexibility of polymers with the bioactivity and ion-release capabilities of MBGs. Mechanical reinforcement and degradation control stem from polymer–glass interactions, enabling applications in bone regeneration, wound healing, and controlled drug delivery. Processing techniques include freeze-drying [87,88], electrospinning, molding, and 3D printing, each providing specific architectural control. Schematics illustrating several typical morphologies of MBG-based structures that can be produced using the synthetic routes discussed above are shown in Figure 5.

## 3. Structure-Directing Agents (SDAs) and Templating Mechanisms

SDAs play a fundamental role in controlling pore size, porosity, and internal nanostructure in MBGs by organizing inorganic precursors around micelles or polymeric assemblies [2,12]. Their ability to form uniformly distributed pores ranging from a few nanometers to several tens of nanometers enables enhanced drug loading and controlled release profiles [3,5]. The type of SDA—whether a nonionic TBC, cationic surfactant, or macromolecular polymer—strongly influences micelle geometry and mesophase symmetry, providing precise control over nanostructural features [14]. The following subsections summarize the major classes of SDAs used for MBG synthesis, their self-assembly behavior, and their effects on mesostructural properties.

### 3.1. Nonionic Triblock Copolymers (TBCs)

**Figure 6:**
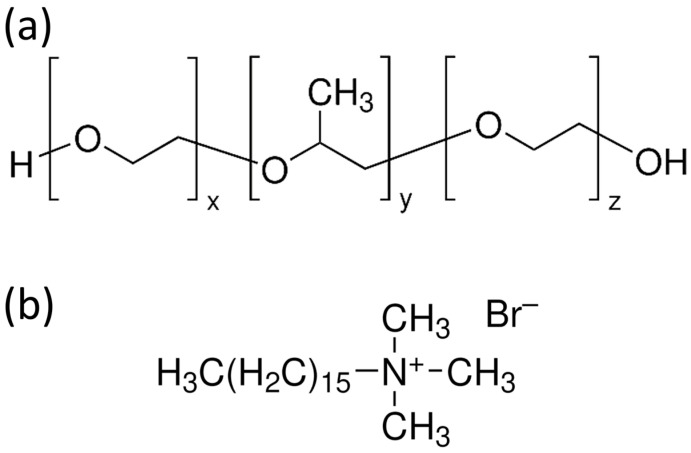
(**a**,**b**) Chemical structures of common Pluronic TBCs (**a**) and CTAB (**b**). Note that for P123, F127, and F68, *x* and *z* are typically equal and stand for 20, 101, and 80 monomers, respectively. Instead, *y* represents 70, 56, and 27 monomers, respectively.

Nonionic TBCs such as Pluronics P123, F127, and F68 (see their chemical structure in Figure 6a) serve as highly effective SDAs due to their amphiphilic EO–PO–EO architecture, which self-assembles in solution to form ordered mesoporous frameworks [2,12]. These copolymers can tune pore diameter, surface area, and mesostructural arrangements by adjusting the hydrophilic–hydrophobic block ratios [2,12]. Their micelles feature hydrophobic PPO cores surrounded by hydrophilic PEO coronas, facilitating the incorporation of Ca and P precursors during TEOS hydrolysis [14]. Pluronic P123 forms worm-like or cylindrical micelles that typically yield 2D hexagonal mesostructures [2]. The mesopores generated by this SDA generally range from 5 to 10 nm, supporting substantial drug-loading capacity and rapid ion-exchange responses [16]. P123-based MBGs possess high surface areas [6,13], which contribute to rapid hydroxyapatite formation and enhanced biological performance [3,5]. Pluronic F127, with a higher EO content than P123, produces more hydrated micelles and often results in smaller mesopores due to stronger EO–silicate interactions [2]. These hydrated micelles form cubic or cylindrical arrangements, generating pore diameters typically between 3 and 6 nm with thicker pore walls [83]. Pluronic F68 forms relatively small micelles because of its shorter poly(propylene oxide) (PPO) block, resulting in smaller pore diameters and more hydrophilic MBG surfaces [2]. Such characteristics influence drug diffusivity and surface chemistry, improving mineralization kinetics under physiological conditions [14]. The templating mechanism follows the classical cooperative self-assembly pathway, wherein hydrolyzed TEOS interacts with the PEO corona, and the inorganic phase condenses around polymer micelles during EISA or sol–gel processing [2,12]. Silicate condensation occurs within the hydrated PEO domain, while Ca–ligand interactions modulate micelle packing and mesophase ordering [16,80].

### 3.2. Cationic Surfactants

Cationic surfactants, particularly CTAB (its chemical structure is depicted in Figure 6b), are widely used in the synthesis of MBGs to create highly uniform and narrowly distributed mesopores [2,12]. CTAB forms positively charged micelles that interact strongly with negatively charged silicate oligomers, promoting the formation of ordered mesoporous structures [2]. The electrostatic interaction between CTAB micelles and hydrolyzed TEOS accelerates inorganic condensation, favoring hexagonal packing and producing pores in the 2–4 nm range [14]. The narrow channels formed by CTAB enhance structural uniformity and enable controlled molecular diffusion [2]. However, CTAB-templated MBGs require careful template removal to eliminate residual toxicity, ensuring complete micelle extraction during calcination or solvent treatment [59].

**Figure 7:**
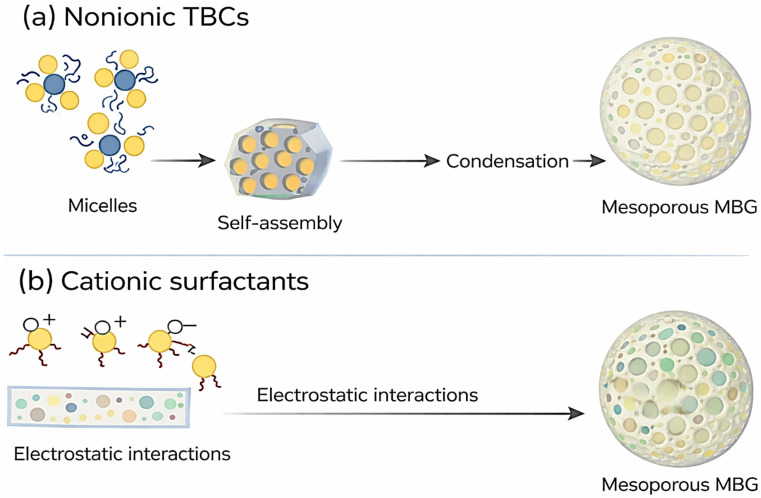
(**a**,**b**) Schematic illustration of templating mechanisms using (**a**) nonionic TBCs and (**b**) cationic surfactants.

Taken together, nonionic triblock copolymers and cationic surfactants represent two fundamentally different structure-directing strategies for tailoring the mesostructure of mesoporous bioactive glasses. While nonionic TBCs rely on cooperative self-assembly between polymer micelles and inorganic species to generate mesophases with relatively larger and more flexible pore architectures, cationic surfactants template mesoporous frameworks primarily through strong electrostatic interactions, resulting in more uniform and narrowly distributed pore systems. These contrasting templating mechanisms and their implications for MBG nanostructure are schematically summarized in Figure 7, highlighting how the choice of SDA directly governs pore organization and mesostructural features.

#### Mechanistic Assessment of Surfactant–Calcium Interactions

In the context of mesoporous bioactive glasses, where calcium incorporation is essential to confer bioactivity, the interaction between Ca^2+^ ions and structure-directing agents plays a decisive role in governing micelle stability, cooperative self-assembly, and the preservation of ordered mesoporosity. The contrasting behavior observed when using non-ionic Pluronic surfactants (P123 and F127) versus cationic cetyltrimethylammonium bromide (CTAB) in calcium-containing systems arises from fundamentally different interaction mechanisms [6,89,90].

Non-ionic triblock copolymers such as P123 and F127 interact with Ca^2+^ primarily through coordination with ether oxygen atoms in their poly(ethylene oxide) (PEO) blocks. These coordination interactions, together with steric stabilization provided by the hydrated PEO corona, mitigate the disruptive effects of increasing Ca^2+^ concentration on micelle integrity. As a result, Pluronic-templated systems can tolerate higher CaO contents while maintaining ordered mesostructures, particularly under acidic synthesis conditions where silicate condensation is slower and better synchronized with template assembly [6,89,90].

In contrast, CTAB relies on electrostatic interactions between its positively charged quaternary ammonium headgroups and anionic silicate species to direct mesostructure formation. In calcium-rich environments, Ca^2+^ ions introduce several destabilizing effects, including charge screening of the CTAB headgroups, competition with CTA^+^ for interaction with negatively charged silicate species, and increased ionic strength of the synthesis medium. These effects reduce electrostatic repulsion between micelles, promote aggregation, and disrupt cooperative self-assembly, leading to loss of long-range order at comparatively lower CaO contents [6,89,90].

The dominant interaction mechanism therefore depends on calcium concentration. At low Ca^2+^ levels, coordination-based stabilization (for Pluronic surfactants) or electrostatic templating (for CTAB) governs mesostructure formation. At intermediate Ca^2+^ concentrations, competition between Ca^2+^–surfactant and Ca^2+^–silicate interactions becomes critical, often resulting in partial loss of mesostructural order. At higher Ca^2+^ concentrations, charge screening, altered solvation, and calcium-rich phase formation dominate, typically preventing the formation of ordered mesoporous structures. These mechanistic differences are summarized in Table 2 and provide a rational basis for selecting appropriate structure-directing agents and synthesis conditions when designing MBGs with elevated CaO contents, as discussed further in Section 4.

### 3.3. Polymeric and Natural SDAs

Various polymers, including PEG, PVA, and PAA, can function as SDAs or co-templates to influence micellar packing, pore size, and silica condensation behavior [42,43,44,45,46]. Their functional groups interact with TEOS-derived silanol species, modifying condensation rates and improving mesopore uniformity. Polymer–inorganic interactions also stabilize the distribution of Ca and P, resulting in more homogeneous mesostructures. PEG-assisted synthesis often produces larger pores by expanding micelle shells or disrupting micelle–micelle interactions [42,43,44,45,46]. Natural polymers such as chitosan, gelatin, and proteins can act as weak SDAs or additives to functionalize pore surfaces, enhance biocompatibility, or modulate degradation behavior [62,63,64,65]. Their amino and hydroxyl groups modify surface chemistry, promoting improved drug adsorption and bioactivity.

### 3.4. Dual-Template and Hierarchical Porosity Strategies

Combining different SDAs enables the creation of hierarchical architectures that integrate mesoporosity with macroporosity, thereby enhancing versatility for biomedical applications [2]. Dual-template systems pair TBCs with CTAB or polymeric macroporogens, producing bimodal mesopore distributions of approximately 4–5 nm and 8–10 nm [83]. The coexistence of multiple pore sizes improves mass transport, promotes cell infiltration, and supports diverse drug-release profiles. Hierarchical pore networks, achieved by combining mesopore templates with sacrificial macropore agents, can generate pores ranging from 100 to 500 µm, which are suitable for tissue engineering scaffolds [2].

### 3.5. Comparative Overview of SDAs

The choice of SDA determines pore diameter, surface area, ordering, and release kinetics (Table 3). Each SDA offers distinct advantages: CTAB produces smaller, well-defined channels; P123 yields larger pores and higher drug-loading capacity; F127 generates more hydrated micelles and thicker pore walls; and F68 increases hydrophilicity and pore uniformity [2,12]. These differences translate into functional benefits, such as enhanced osteoinductivity, mechanical stability, or tailored ion release [14,16,83]. Collectively, these SDAs provide a versatile toolbox for designing MBGs with precise structural, chemical, and functional attributes tailored to a wide range of biomedical applications [2,12].

## 4. Influence of Composition and Therapeutic Ion Doping

### 4.1. Effects of Base Composition on Mesostructure and Bioactivity

MBGs are commonly formulated within the SiO_2_–CaO–P_2_O_5_ ternary system, where silica acts as the primary network former, calcium serves as a network modifier influencing dissolution, and phosphorus contributes to biomineralization through phosphate release [3,5]. The balance among these oxides governs mesophase stability, pore ordering and degradation kinetics, making compositional design a critical parameter in MBG development [15,16,17]. During sol–gel processing and cooperative self-assembly, these compositional ratios also influence the interactions between inorganic species and surfactant micelles, thereby linking base composition to mesostructure formation.

#### 4.1.1. Calcium Oxide Content: Context-Dependent Effects on Mesostructure and Bioactivity

Among the base compositional parameters of mesoporous bioactive glasses, the CaO content plays a central role in regulating bioactivity, dissolution behavior, and hydroxyapatite formation. At the same time, CaO strongly influences mesostructural stability through its function as a network modifier. Importantly, however, the relationship between CaO content and mesopore ordering does not follow rigid or universal compositional thresholds but instead reflects the combined effects of composition and synthesis conditions.

Calcium ions disrupt Si–O–Si linkages in the silicate network, generating non-bridging oxygens (NBOs) and reducing network connectivity, which accelerates glass dissolution and enhances bioactivity [17,91]. This effect is commonly rationalized using the concept of network connectivity, which decreases with increasing CaO content and correlates with progressive depolymerization of the silicate framework. As CaO content increases, the glass network becomes increasingly fragmented, rendering cooperative self-assembly between inorganic species and structure-directing agents more sensitive to processing conditions.

**Figure 8:**
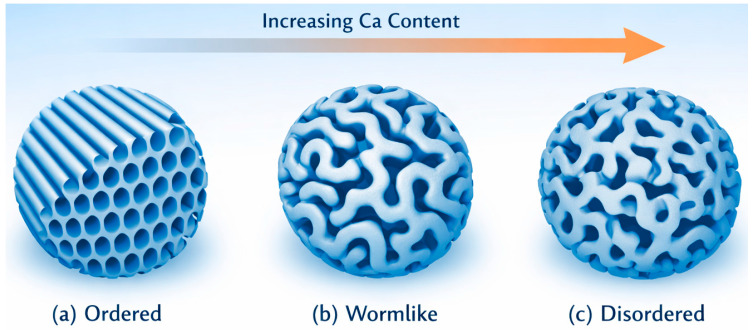
Illustration of the influence of Ca content on the MBG mesopore ordering. With increasing the Ca content, the morphology evolves from ordered (**a**) to wormlike (**b**), to disordered (**c**) MBGs.

Numerous experimental studies report that increasing CaO content is generally associated with a transition from highly ordered hexagonal mesoporous structures to worm-like or disordered pore architectures, accompanied by a reduction in BET surface area [17,89,92,93]. This behavior is schematically illustrated in Figure 8, which shows the representative evolution of mesostructure with increasing CaO content under typical synthesis conditions. While such observations have led to frequently cited CaO “thresholds” (often in the range of ~15–20 mol%), these values should be interpreted as system-dependent trends rather than absolute limits.

The effective CaO range compatible with ordered mesoporosity is strongly influenced by synthesis parameters. The choice of structure-directing agent is particularly critical: non-ionic triblock copolymers such as P123 or F127 can stabilize ordered mesostructures at higher CaO contents through coordination-based interactions between Ca^2+^ ions and poly(ethylene oxide) blocks, whereas CTAB-templated systems typically exhibit mesostructural degradation at lower CaO contents due to electrostatic screening and ionic-strength effects [89]. Solution pH further modulates silicate condensation kinetics and Ca^2+^ speciation, with acidic conditions favoring controlled assembly at elevated CaO contents, while neutral or basic conditions accelerate condensation and exacerbate Ca^2+^-induced disruption [19,94].

Thermal treatment parameters, including heating rate, calcination temperature, and dwell time, also play a decisive role in determining whether mesoporous architectures are preserved in Ca-rich compositions. Reduced network rigidity at higher CaO contents increases susceptibility to pore collapse or partial crystallization during template removal, particularly under rapid heating or high calcination temperatures [95].

Taken together, these observations demonstrate that CaO content cannot be considered in isolation when evaluating mesostructural stability in MBGs. Instead, the apparent CaO “threshold” for maintaining ordered mesoporosity emerges from the interplay between base composition and synthesis conditions. By optimizing surfactant selection, solution chemistry, and thermal treatment, it is possible to extend the practical CaO range while preserving beneficial mesoporous characteristics, thereby enabling the rational design of MBGs that balance high bioactivity with structural integrity for biomedical applications.

#### 4.1.2. Role of P_2_O_5_ Content in Ternary MBG Systems

Phosphorus pentoxide (P_2_O_5_) plays a distinct yet complementary role in ternary SiO_2_–CaO–P_2_O_5_ mesoporous bioactive glasses by directly influencing bioactivity, degradation behavior, and ion-release kinetics. Unlike CaO, which primarily acts as a network modifier, phosphate species can function either as network formers or as isolated orthophosphate units depending on composition and synthesis conditions, thereby exerting a nuanced influence on mesostructure and biological response.

At moderate P_2_O_5_ contents (typically ≤10 mol%), phosphate groups are predominantly incorporated as isolated orthophosphate or short-chain pyrophosphate units within the silicate matrix, promoting rapid hydroxyapatite nucleation while largely preserving mesoporous order [89,96]. In this compositional regime, P_2_O_5_ enhances surface reactivity without substantially disrupting cooperative surfactant-directed assembly.

Increasing P_2_O_5_ content accelerates network depolymerization and can compete with silica condensation during sol–gel processing, particularly in acidic environments where phosphate species remain highly mobile. As a result, phosphate-rich MBGs often exhibit reduced long-range mesostructural ordering, lower BET surface areas, and increased dissolution rates [17,97]. These effects become more pronounced when high P_2_O_5_ contents are combined with elevated CaO levels, highlighting the coupled nature of compositional parameters in ternary systems.

From a functional perspective, phosphate enrichment can be advantageous for applications requiring rapid bioactivity or fast ion release, such as early-stage bone regeneration. However, this benefit is often achieved at the expense of mesoporous structural stability, underscoring a trade-off between biological performance and pore ordering. Consequently, the influence of P_2_O_5_ content must be interpreted in conjunction with CaO concentration and synthesis parameters, rather than as an independent variable.

Overall, the role of P_2_O_5_ in MBGs reinforces the importance of considering base-composition effects within the full SiO_2_–CaO–P_2_O_5_ ternary framework. Rational compositional design therefore requires balancing phosphate-driven bioactivity against mesostructural preservation, guided by both compositional ratios and processing conditions. Taken together, these composition-driven effects demonstrate how changes in base glass chemistry translate into differences in mesostructural stability, dissolution behavior, and ultimately biological performance, thereby linking compositional design directly to application-specific requirements.

### 4.2. Effects of Therapeutic Ion Doping

**Figure 9:**
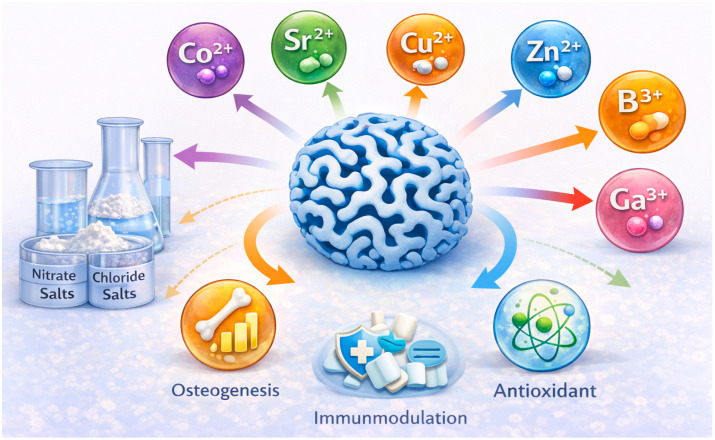
Schematics of a therapeutic ion-doped MBG nanoparticle.

Incorporating therapeutic ions into MBGs (Figure 9) imparts functionalities such as osteogenesis, angiogenesis, antibacterial activity, immunomodulation, and antioxidant effects. These ions are typically introduced during sol–gel synthesis in the form of nitrate or chloride salts. However, ions susceptible to thermal reduction (e.g., Ag^+^, Cu^2+^) often require post-synthetic incorporation [20,28,80]. Dopant concentrations must be carefully controlled to maintain mesophase ordering and prevent crystallization.

Therapeutic ions modify network depolymerization, influence ion-release kinetics, and regulate cellular responses, enabling MBGs to be tailored for diverse biomedical applications [20,21,38] (Table 4). Strontium (Sr^2+^) can be incorporated up to 10 mol% SrO without loss of mesopore order; it enhances osteogenesis, suppresses osteoclast activity, and accelerates HA formation [4,5,80,85]. Copper (Cu^2+^), typically at 1–5 mol% CuO, provides antibacterial and angiogenic properties and inhibits pathogens including Staphylococcus aureus and Escherichia coli [12,28,58,77,86]. Zinc (Zn^2+^), at 1–5 mol% ZnO, supports osteogenesis, antibacterial activity, and wound healing, with evidence of enhanced angiogenesis in vivo [21,25,78,98,99,100]. Boron (B^3+^), incorporated at ≤5 mol% B_2_O_3_, improves osteogenesis, angiogenesis, and network connectivity [35,55,59,99]. Cerium (Ce^3+^/Ce^4+^), incorporated at 1–5 mol% CeO_2_, offers antioxidant and anti-inflammatory effects and promotes osteogenic differentiation under oxidative stress [79,81,101,102,103]. Beyond cerium, other lanthanide ions have attracted growing interest in MBG systems. Europium (Eu^3+^) has been primarily explored as a luminescent probe for bioimaging and material tracking, enabling combined therapeutic and diagnostic functionality [104,105]. Samarium (Sm^3+^) and lanthanum (La^3+^) have been reported to influence osteogenic differentiation and antibacterial behavior, while also modifying glass network connectivity and degradation kinetics [106,107]. Additional ions include cobalt (Co^2+^, angiogenic, 1–3 mol% CoO) [20,31], gallium (Ga^3+^, antibacterial and immunomodulatory, 1–3 mol% Ga_2_O_3_) [108,109], manganese (Mn^2+^, osteogenic, 1–3 mol% MnO) [25,73], iron (Fe^3+^, magnetic for hyperthermia, 5–10 mol% Fe_2_O_3_) [57], and magnesium (Mg^2+^, osteogenic and cardioprotective) [3,110]. Compositional tuning—through adjusting base oxide ratios and incorporating therapeutic ions—enables precise control of MBG degradation, ion release, mesoporosity, and biological performance [38]. Tailoring these parameters provides a powerful strategy for engineering MBGs suitable for bone regeneration, wound healing, antibacterial applications, angiogenesis, and antioxidant therapies.

## 5. Characterization Techniques for MBGs

MBG research utilizes a consistent set of physicochemical and biological assays to characterize structure, composition, texture, bioactivity, and cytocompatibility, enabling correlations between synthesis variables (such as polymer type and templating conditions) and material performance [83,111]. Common characterization methods—including XRD, FTIR, BET/N_2_ adsorption, TEM, SEM, ICP-OES, and SBF mineralization tests—are routinely employed to evaluate mesostructure, surface area, ion release, and apatite formation, facilitating comparisons among polymer-assisted synthesis routes [83,111]. Quantitative surface area and pore volume data reported across studies highlight how templating approaches influence textural properties [100,112]. These complementary techniques are essential for establishing relationships among mesostructural order, composition, and biological activity (Table 5).

### 5.1. X-Ray Diffraction (XRD)

XRD is a crucial characterization technique for MBGs because it provides essential information about their amorphous structure, mesoscopic order, and the formation of crystalline phases following synthesis or ion doping [10,83,111]. Amorphous MBGs typically exhibit a broad diffuse halo in wide-angle XRD patterns, indicating the absence of long-range order, which is vital for high ion-release rates and bioactivity. Wide-angle XRD is routinely employed in MBG research to verify that calcination temperatures (typically 600–700 °C for silicate MBGs) do not induce unwanted crystallization, which would diminish bioactivity and surface reactivity [59,81]. Low-angle XRD is used to detect ordered mesoporous structures in MBGs synthesized via surfactant-templated sol–gel or EISA methods, revealing reflections such as (100), (110), and (200) corresponding to hexagonal or cubic pore arrangements [1,2]. These reflections indicate the degree of mesophase ordering, where sharp and intense peaks correspond to highly ordered P123- or F127-templated MBGs, while broadened peaks suggest worm-like or partially ordered mesostructures [2,14]. XRD is also applied to monitor unwanted crystallization caused by dopants such as Ca^2+^, Cu^2+^, Zn^2+^, or Sr^2+^, and to detect crystalline HA peaks that emerge after SBF immersion during bioactivity testing [27,83,111].

### 5.2. Transmission Electron Microscopy (TEM)

TEM plays a central role in the characterization of MBGs, providing direct nanoscale visualization of pore arrangements, particle morphology, and structural order [6,40,79,109]. TEM can reveal hexagonal, cubic, or worm-like pore architectures, confirm uniform pore distribution, and provide particle size information for nanoparticles synthesized via sol–gel or modified Stöber methods [3,6,79,109]. TEM images often validate the mesostructural integrity after calcination, confirming that surfactant removal did not collapse pore channels or induce crystallization [16]. Doped MBGs can be analyzed by TEM to identify core–shell structures or elemental distribution, while energy-dispersive X-ray spectroscopy (EDX)-equipped TEM enables nanometer-scale mapping of dopants such as Si, Ca, P, Cu, Zn, or Sr [3,5]. High-resolution TEM (HRTEM) and selected area electron diffraction (SAED) are frequently used to verify that samples remain amorphous after heat treatment, with diffuse rings indicating the retention of a glassy structure [59]. Despite its high resolution, TEM requires demanding sample preparation and is limited by small sampling volume, beam-induced damage, and challenges in analyzing hydrated or organic hybrid materials.

### 5.3. Scanning Electron Microscopy (SEM)

SEM complements TEM by visualizing surface morphology, particle shape, aggregation state, and scaffold macroporosity [10,14,15,35,109]. SEM identifies particle geometry (spherical, irregular, rod-like), assesses surface roughness, and evaluates morphological changes following calcination or polymer compositing. It is particularly valuable in MBG-polymer composites, where it reveals pore interconnectivity, coating uniformity, and the MBG–polymer interface in scaffolds produced by freeze-drying, electrospinning, or 3D printing [56]. After SBF immersion, SEM visualizes the formation of apatite layers, illustrating the transition from smooth MBG surfaces to needle-like HA crystals. SEM combined with EDX enables semi-quantitative elemental analysis during bioactivity tests, verifying Ca/P ratios, dopant distribution, and ion-exchange behavior [36,81]. Often, with SEM or TEM, energy-dispersive X-ray spectroscopy (EDS/EDX) is used to determine the elemental composition and distribution within MBGs. It confirms the stoichiometry of synthesized MBGs, maps ion distribution, and verifies any phase separation or dopant clustering within the material [4,13,14,20,80].

### 5.4. Fourier-Transform Infrared Spectroscopy (FTIR)

FT-IR provides essential chemical information by detecting Si–O–Si network vibrations, silanol groups, phosphate modes, carbonates, and potential organic residues [10,15,40,79]. Characteristic FTIR bands such as Si–O–Si stretching vibrations (~1050–1100 cm^−1^), Si–OH-related features (~950 cm^−1^), and P–O bending modes (~560–600 cm^−1^) are commonly used to evaluate network connectivity and confirm the incorporation of calcium and phosphorus species in mesoporous bioactive glasses [97,113]. However, in multicomponent SiO_2_–CaO–P_2_O_5_ systems, the absorption band observed in the ~950 cm^−1^ region cannot be unambiguously assigned to silanol (Si–OH) groups alone. As reported in the literature, this spectral region contains overlapping contributions from Si–O^−^ stretching vibrations associated with non-bridging oxygens, Si–OH groups, and P–O stretching modes from phosphate species, which complicates direct interpretation. Consequently, FTIR analysis in this region should be interpreted qualitatively and in conjunction with other spectral features, while definitive assessment of network connectivity and phosphate incorporation requires complementary techniques such as solid-state ^29^Si and ^31^P NMR spectroscopy [113,114]. FTIR is widely used to verify surfactant removal after calcination by monitoring the disappearance of C–H stretching bands from P123, F127, or CTAB templates [16]. Additionally, FTIR confirms HA formation after SBF exposure through the emergence of phosphate and hydroxyl bands, enabling rapid assessment of bioactivity [3,5,14]. In polymer–MBG composites, FTIR detects functional bonds (e.g., C=O, N–H, C–O–C) associated with chitosan, GelMA, PEG, or PVA, confirming successful grafting or blending [35,59]. Although powerful, FTIR provides qualitative rather than quantitative data and may suffer from overlapping peaks that complicate interpretation.

### 5.5. Inductively Coupled Plasma Optical Emission Spectroscopy (ICP-OES)

ICP-OES is essential for determining elemental composition, dopant incorporation efficiency, and ion-release kinetics during dissolution studies [14,15,55,115]. ICP-OES quantifies elements such as Si, Ca, P, Na, Mg, Sr, Zn, Cu, Fe, and others with high sensitivity and is widely used to analyze dissolution in SBF and physiological media. It is also the preferred technique for monitoring therapeutic ion release from doped MBGs (e.g., Sr^2+^, Zn^2+^, Cu^2+^), correlating cumulative ion release with osteogenic, antibacterial, or angiogenic responses [28,98]. Although this method is destructive and does not provide bonding or structural information, its precision and wide dynamic range make it indispensable.

### 5.6. The Brunauer–Emmett–Teller (BET) Method

The BET method is essential for evaluating surface area, pore size, and pore volume, which directly influence MBG reactivity, drug-loading capacity, and ion-release behavior [14,28,35,100]. Nitrogen adsorption–desorption isotherms reveal textural characteristics such as type IV behavior and hysteresis loops associated with mesopores. BET is widely used to compare templated and non-templated MBGs, confirming the large surface areas (200–700 m^2^/g) and narrow pore size distributions produced by P123- or F127-assisted synthesis [14]. BET can also track structural modifications after doping or polymer functionalization, which often reduce pore accessibility. Successful removal of the organic template is typically marked by a sharp increase in surface area and pore volume after calcination [16]. Limitations include the requirement for dry, degassed samples and the inability to provide chemical information. Bioactivity is commonly assessed through simulated body fluid (SBF) immersion tests, which monitor HA formation on MBG surfaces using XRD, FTIR, and SEM [3,4,5]. SBF tests routinely show accelerated HA growth in doped MBGs (e.g., Sr- or Zn-containing glasses), correlating with enhanced osteogenic responses [5,85]. Ion-release studies conducted by ICP-OES complement SBF tests and help correlate dissolution profiles with cellular responses. Biological assays—including cell viability, ALP activity, and osteogenic gene expression—establish cytocompatibility and confirm the functional benefits of doped MBGs [21,78].

### 5.7. Additional Characterization Techniques

Other characterization techniques capable of providing either compositional or structural analysis, as well as evaluating the biological response of MBGs, are worth mentioning. The first category includes nuclear magnetic resonance (NMR). For example, solid-state NMR (e.g., ^29^Si, ^11^B, ^31^P NMR) offers detailed information about the local atomic arrangement, network structure, and connectivity. Specifically, ^29^Si NMR distinguishes different Q^n^ species (Q^2^, Q^3^, Q^4^), ^11^B NMR differentiates BO_3_ and BO_4_ units in borate glasses, and ^31^P NMR characterizes the phosphate environment [59]. This technique is particularly valuable for understanding subtle changes in the glass network structure due to doping or processing [59]. The second category includes techniques such as SBF immersion, which evaluates the in vitro bioactivity of MBGs by mimicking physiological temperature and pH conditions to determine whether an HA layer forms on the MBG surface [16,80]; cell viability and proliferation assays, which use common cell lines like osteoblasts, mesenchymal stem cells, fibroblasts, and cancer cells, assessed through metabolic activity, live/dead staining, or DNA quantification [16,57,80]; osteogenic differentiation, evaluated using various markers [80,85,100]; antibacterial activity, assessed against organisms such as Staphylococcus aureus, Escherichia coli, and Pseudomonas aeruginosa using methods like disk diffusion assays, colony counting, or live/dead bacterial staining [1,12,25,73,74,75,86]; and in vivo studies based on rat, rabbit, and sheep models, which are used to evaluate bone regeneration, vascularization, and immune response [5,98].

## 6. Biomedical Applications of MBGs

**Figure 10:**
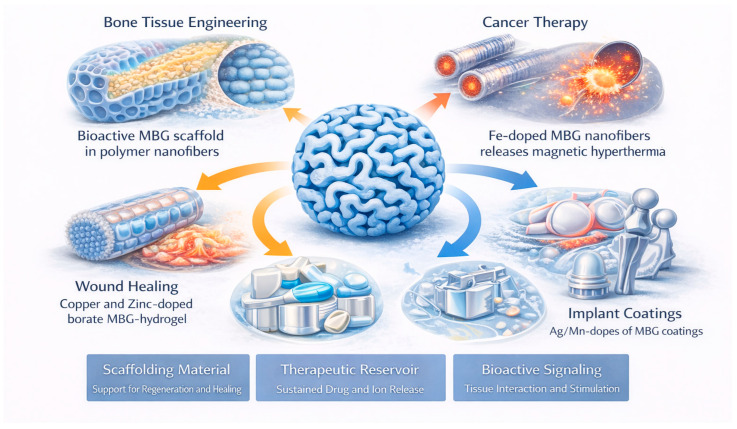
Schematics showing the main biomedical applications of MBGs.

MBGs possess a unique combination of a large surface area, tunable pore architecture, compositional versatility, and intrinsic bioactivity, enabling their integration across a wide range of biomedical applications (Figure 10). Their ordered mesopores facilitate drug loading and sustained release, while their Si–Ca–P network interacts favorably with physiological environments by forming hydroxyapatite and releasing ions that promote tissue regeneration [2,6,10,14,15,30]. These complementary structural and chemical features allow MBGs to function simultaneously as scaffolding materials, therapeutic reservoirs, and bioactive signaling platforms (Table 6).

Accordingly, the selection of appropriate MBG compositions and architectures requires balancing mesostructural features, mechanical integrity, and ion release behavior.

### Structure–Property–Application Trade-Offs in Mesoporous Bioactive Glasses

The design of mesoporous bioactive glasses requires careful balancing of mesostructural features, mechanical integrity, and ion release behavior, as these parameters are intrinsically interdependent and strongly influence application performance [93,116]. While highly ordered mesoporosity and high specific surface area are often desirable for enhanced bioactivity and drug-loading capacity, these features can compromise mechanical stability and long-term structural integrity, particularly at elevated calcium contents [117].

Increasing mesoporosity and pore volume generally enhances dissolution kinetics and therapeutic ion release due to greater accessible surface area [118]. However, highly porous MBGs typically exhibit reduced mechanical strength and fracture resistance, limiting their suitability for load-bearing applications. Conversely, partial densification or reduced pore ordering can improve mechanical stability but may slow ion release and reduce bioactivity [17,119]. These opposing trends highlight the necessity of tailoring MBG structure to the specific functional requirements of the target application.

In composite systems, such as polymer–MBG hybrids, these trade-offs can be partially mitigated. Polymer infiltration or surface functionalization has been shown to enhance mechanical performance and toughness while preserving mesoporosity and controlled ion release [119]. The extent of polymer penetration, interfacial bonding, and spatial distribution of the inorganic phase play critical roles in determining the overall performance of the composite system.

Application-driven optimization is therefore essential. For bone regeneration in non-load-bearing or minimally loaded sites, highly mesoporous MBGs with rapid ion release may be advantageous to accelerate osteogenesis and biomineralization. In contrast, applications requiring greater mechanical stability, such as scaffolds subjected to moderate load or long-term implantation, may benefit from less ordered mesostructures, lower porosity, or composite architectures that balance bioactivity with structural durability. Explicit recognition of these structure–property–application trade-offs enables more rational MBG design and avoids one-size-fits-all strategies that may be unsuitable for specific clinical contexts [6,120].

In *bone tissue engineering*, as an example of these structure–property trade-offs, MBGs promote osteogenesis through rapid ion exchange, surface reactivity, and the formation of bioactive layers that support cell adhesion and differentiation [6,27,31,32]. To overcome the brittleness inherent in pure glasses, polymer–MBG composites incorporating PCL, PLA, PVA, PHBV, chitosan, gelatin, or zein form flexible, mechanically reinforced structures suitable for load-bearing or structurally demanding sites [33,34,40,60,73,121]. These composites can be fabricated as nanofiber mats, aerogels, foams, or patient-specific 3D-printed scaffolds, each providing controlled macroporosity and interconnected networks that facilitate nutrient transport and vascular infiltration [1,55,56,62]. The incorporation of therapeutic ions—including Sr^2+^, Cu^2+^, Zn^2+^, and B^3+^—further enhances cellular responses by stimulating osteogenesis, angiogenesis, and antibacterial protection in bone defects [14,20,26,37,77]. These synergistic effects make MBGs particularly suitable for regenerative strategies requiring both structural support and biofunctional stimulation. Their mesostructure renders MBGs highly efficient drug carriers, enabling controlled release profiles for anticancer, antibiotic, anti-inflammatory, or antioxidant therapeutics [83,111]. Loading drugs within uniform mesopores provides high capacity and sustained delivery, while polymer–MBG matrices can further modulate release kinetics. Applications range from localized chemotherapy—where Fe-doped MBGs support combined magnetic hyperthermia and drug transport—to infection control using gentamicin-loaded MBGs that maintain antibacterial activity over extended periods [57,101,122]. The tunability of pore size, surface chemistry, and degradation rate allows drug delivery systems to be adapted for systemic, topical, or implant-associated release.

In *wound healing*, particularly in chronic or diabetic wounds, MBGs and mesoporous borate formulations promote tissue repair by releasing ions that accelerate angiogenesis, fibroblast migration, and extracellular matrix deposition [31,36]. Their antibacterial properties—enhanced by dopants such as Cu^2+^ or Zn^2+^—help control infection, while hydrogel–MBG systems create moist, bioactive environments that facilitate regeneration [76]. These multifunctional properties support rapid wound closure and reduce inflammation, effectively addressing challenges commonly encountered in impaired healing conditions.

*Dental applications* also benefit from the reactivity and ion release profile of MBGs. Their ability to remineralize enamel, inhibit bacterial growth, and reinforce dental composites has expanded their use in restorative dentistry and caries management. Incorporating MBG nanospheres into resins preserves mechanical properties while providing antibiofilm activity and supporting remineralization through sustained Ca and P release [77,86]. When combined with amorphous calcium phosphate or cerium-containing nanoparticles, MBGs promote the formation of protective fluorapatite layers and strengthen early-stage carious lesions [67,79]. These developments illustrate the growing relevance of MBGs in preventive and restorative dental care.

Beyond the musculoskeletal and dental fields, MBGs have demonstrated therapeutic potential in *cardiovascular medicine*, *soft tissue regeneration*, and *hemostasis*. Magnesium-containing MBGs loaded with gallic acid exhibit cardioprotective effects in ischemia–reperfusion injury by reducing inflammation and enhancing functional recovery [110]. Injectable MBG–alginate hydrogels incorporating melatonin support intervertebral disk regeneration by preserving disk height and modulating inflammatory pathways [123]. In acute bleeding scenarios, MBG-loaded cryogels containing copper ions achieve rapid blood coagulation and provide antimicrobial protection, making them promising hemostatic agents [65]. Surface coatings based on Ag-, Mn-, or Sr-modified MBGs improve the osseointegration and antibacterial performance of titanium, stainless steel, or PEEK implants [25,58,73,75]. These examples illustrate the versatility of MBGs in complex clinical environments that require both structural integration and therapeutic modulation.

While in vitro studies provide essential mechanistic insights into MBG bioactivity, ion release behavior, and cell–material interactions, their predictive value for in vivo performance remains limited. In vivo outcomes are influenced by additional factors such as immune response, vascularization, mechanical loading, and the local biological environment, which are not fully captured in simplified in vitro models [124]. Reported discrepancies between promising in vitro results and more variable in vivo outcomes highlight the need for cautious interpretation and application-driven validation [125]. Consequently, in vitro findings should be regarded as an initial screening step that must be complemented by well-designed in vivo studies to ensure translational relevance.

**Table 6:** Overview of MBG applications and key findings.

Application Area	Key MBG Feature Utilized	Specific Example/Study	Key Finding/Benefit	Refs.
Bone tissue engineering	bioactivity, porosity, polymer composites	Sr-MBG/PVA composite scaffolds	enhanced bioactivity and osteogenic differentiation	[4]
	hierarchical porosity (macro- + mesopores)	3D-printed MBG scaffolds with AgNPs	antibacterial, osteoblast proliferation, bone formation	[1]
	ion doping (e.g., Sr, Zn, B)	Zn-MBGs in sheep model	promoted bone regeneration, angiogenesis, osteogenesis	[98]
Drug delivery (anticancer)	high surface area, mesopores, sustained release	Silibinin-releasing MBG nanoparticles	cytotoxic to breast cancer cells, sustained release	[74]
	magnetic properties (Fe-doped MBGs)	Fe-doped MBG nanofibers for melanoma therapy	magnetic hyperthermia, >80% cell death	[57]
Drug delivery (antibiotic)	high surface area, mesopores, controlled release	gentamicin-loaded MBGs	effective antibacterial activity, sustained release over 10 days	[122]
	dual functionality (antibiotic + antioxidant)	Ce-MBG scaffolds loaded with gentamicin	antibiotic delivery, antioxidant properties	[101]
Wound healing	bioactivity, angiogenesis, antibacterial, degradation	Zn-/Cu-doped borate MBGs	accelerated wound closure, enhanced angiogenesis, antibacterial activity	[21]
	antioxidant, antibacterial, pro-healing (in hydrogels)	Cu-doped MBG nanozyme cryogels	accelerated diabetic wound closure, reduced inflammation	[76]
Dental applications	bioactivity, remineralization, antibacterial	Cu-doped MBG nanospheres in dental composites	maintained mechanical properties, antibacterial activity against *S. mutans*	[77,86]
	enamel remineralization (with ACP)	MBGs loaded with amorphous calcium phosphate	significant enamel remineralization, fluorapatite layer formation	[67]
Cardiovascular applications	cardioprotective, antioxidant	Mg-doped MBGs with gallic acid	reduced infarct size, improved cardiac function, anti-inflammatory	[110]
Intervertebral disk regeneration	anti-inflammatory, ECM synthesis promotion, injectability	MBG/sodium alginate hydrogel with melatonin	preserved disk height, reduced inflammation, promotes ECM synthesis	[123]
Hemostatic applications	rapid coagulation, antibacterial	Cu-ion loaded MBGs in chitosan/gelatin cryogels	rapid hemostasis, effective for acute/persistent bleeding	[65]
Coatings for implants	bioactivity, osseointegration, antibacterial	Ag/Mn-doped MBG coatings on PEEK	enhanced osteogenic differentiation, antibacterial activity	[73]
	bioactivity, osseointegration, long-term stability	zein/Ag-Sr doped MBG coatings on titanium	antibacterial, osteogenic, uniform coating	[58,75]

## 7. Challenges and Future Perspectives

### 7.1. Current Challenges

MBGs depend on finely balanced sol–gel reactions and polymer-mediated self-assembly, which are highly sensitive to processing variables such as pH, evaporation rate, and precursor reactivity. This sensitivity complicates scale-up and can limit reproducibility when transitioning beyond laboratory conditions [83,111]. These challenges are directly linked to the compositional and structural limitations observed in MBGs synthesized via classical sol–gel methods. Scalability remains a major obstacle, as maintaining structural order and batch consistency becomes difficult when production is scaled from benchtop to industrial levels [14,126]. High CaO (>25 mol%) or P_2_O_5_ (>40 mol%) contents can disrupt the cooperative assembly of silica with surfactant micelles, leading to mesophase collapse or disordered pore formation [14,17]. Additionally, the calcination step—typically confined to a narrow temperature range of 600–700 °C for silicate MBGs—is highly sensitive; insufficient heating leaves residual organics, while excessive temperature causes crystallization or pore collapse [59,81].

In polymer-templated systems, micelle stability is crucial for the successful formation of ordered mesostructures and can be disrupted by dopants, solvent composition, or temperature fluctuations [6,14,80]. These structural challenges translate into practical difficulties in developing mechanically robust and clinically viable scaffolds. Pure MBGs are brittle, requiring reinforcement with polymeric matrices to achieve sufficient toughness and flexibility for bone or load-bearing applications [29,30]. Controlling degradation kinetics remains equally challenging: MBGs may degrade either too quickly or too slowly relative to tissue healing rates, and ion release can exhibit undesirable burst profiles [17,24,115]. Shelf-life issues have also been reported; prolonged storage can lead to silanol condensation and surface area loss, thereby reducing bioactivity [126].

Biological performance further depends on balancing rapid dissolution, therapeutic ion release, and the avoidance of inflammatory responses, particularly for compositions containing antibacterial ions such as Ag^+^ or Cu^2+^ [15,40]. In vivo responses amplify these concerns and underscore the remaining barriers to clinical application. Differences between in vitro and in vivo degradation complicate predictions of long-term behavior [17]. Certain dopants can provoke inflammatory responses if their concentrations are not precisely controlled [1,28]. Large scaffolds often suffer from insufficient vascularization in central regions, which compromises tissue integration [5]. Clinically, MBGs face slow regulatory progress, high synthesis costs, and limited translation into large-scale clinical trials [6,10].

### 7.2. Future Research Directions

Advances in polymer chemistry, templating strategies, and multiscale manufacturing present promising opportunities to overcome current limitations and enable next-generation MBG systems tailored for precision medicine [47,127,128]. These emerging approaches (Table 7) align well with the compositional and architectural insights established by contemporary MBG research. Eco-friendly sol–gel methods employing biobased surfactants, plant extracts, or reduced-solvent syntheses aim to minimize environmental impact while maintaining mesostructural control [111]. Precision processing techniques—including aerosol-assisted spray drying, microfluidics, and multi-material 3D printing—offer enhanced morphological control and patient-specific scaffold architectures [1,29,56]. Real-time in situ methods, such as synchrotron SAXS are expected to refine the mechanistic understanding of EISA and guide optimization of mesophase formation [59]. Computational modeling, including molecular dynamics simulations, is progressively shaping predictive design frameworks for MBG networks and dopant interactions [14].

Functional diversification remains a promising future direction: stimuli-responsive MBGs capable of pH-triggered release, magnetic activation, enzymatic responsiveness, or photothermal conversion are expected to enable adaptive implants and smart therapeutic platforms [115]. These concepts naturally extend into broader biomedical contexts. Personalized medicine approaches utilizing CT/MRI-based 3D printing, tailored ion doping, and integration with stem cells aim to create patient-specific grafts with enhanced regenerative capacity [29,56]. Future applications include neural regeneration, myocardial repair, advanced cancer therapies, and biosensing technologies [47,50,57,74,110]. Advanced composites incorporating 2D materials such as graphene or MXenes promise substantial improvements in mechanical strength, electrical conductivity, interfacial bonding, and multifunctionality [29,30].

Looking ahead, further progress in MBG research will require closer integration of fundamental understanding with translational considerations. Key challenges include improving the predictive correlation between in vitro and in vivo performance, ensuring reproducibility and scalability of synthesis routes, and defining safe and effective therapeutic ion dosage windows [6]. Advances in computational modeling, data-driven materials design, and standardized in vivo evaluation protocols are expected to play an increasingly important role in accelerating clinical translation. Addressing these challenges will be essential for moving MBGs from promising laboratory materials toward reliable and clinically applicable technologies.

## 8. Conclusions

MBGs represent a pivotal advancement in biomaterials science, combining the inherent bioactivity of traditional glass systems with the structural precision and high surface area characteristic of mesoporous materials. This combination enables finely controlled interactions with biological environments and supports a broad range of therapeutic applications.

Throughout their development, the defining characteristics of MBGs arise from the interplay among synthesis strategy, structural design, and biological function. The architecture of MBGs is directed by structure-directing agents such as TBCs and cationic surfactants, which facilitate the self-assembly of highly ordered pore networks. Achieving an optimal mesostructure depends on mastering key synthesis parameters—SDA concentration, pH, aging conditions, and calcination protocols—each of which directly influences pore order, network connectivity, and chemical homogeneity. The controlled incorporation of therapeutic ions further enhances MBG functionality, enabling improved osteogenesis, angiogenesis, antibacterial activity, and anti-inflammatory effects.

MBGs are chemically and structurally tunable systems supported by a robust toolbox of characterization techniques that link nanoscale architecture to macroscopic performance, forming the basis for their optimization across biomedical applications. Such a comprehensive characterization framework reinforces the strong correlation between MBG design and functional outcomes. Advanced analytical techniques—including TEM, SEM, XRD, FTIR, NMR, SAXS, BET, and ICP-OES—provide deep insights into mesopore symmetry, chemical bonding, elemental distribution, and ion-release behavior. This detailed understanding facilitates the successful application of MBGs in bone regeneration through composite and hierarchical scaffolds, drug delivery systems for anticancer and antibiotic therapies, wound-healing strategies, dental treatments, cardiovascular approaches, and emerging fields such as intervertebral disk repair.

Despite these achievements, several challenges continue to influence the trajectory of MBG research, particularly those related to scale-up, reproducibility, degradation dynamics, and biological integration. These persistent barriers underscore the need for next-generation approaches that combine precision, sustainability, and clinical adaptability. Future progress will depend on adopting greener synthesis methods, refining advanced manufacturing techniques such as aerosol-assisted processing and multi-material 3D printing, and developing intelligent MBGs capable of stimuli-responsive or multifunctional behavior. Personalized scaffolds that incorporate tailored ion doping and advanced composite designs offer promising avenues to address individual patient needs, while interdisciplinary strategies will accelerate the translation of MBGs into practical clinical solutions. Collectively, these advancements position MBGs as one of the most versatile and forward-looking platforms in regenerative medicine and therapeutic delivery. By uniting structural precision with biological functionality, MBGs are poised to make significant contributions to future biomedical innovation. As challenges are systematically addressed and emerging technologies continue to evolve, mesoporous bioactive glasses are expected to enable a new generation of regenerative and therapeutic interventions—advancing human health and profoundly expanding the capabilities of modern biomaterials.

## Figures and Tables

**Table 1:** Critical synthesis parameters and their influence on MBG structure and properties.

Parameter	Optimal Range/Condition (for Silicate MBGs)	Effect of Deviation (Examples)	Impact on MBG Properties	Refs.
TEOS/SDA molar ratio	10–40 (for P123)	higher ratios	increased pore ordering, but may reduce pore volume	[14]
pH and catalyst type	pH 1–2 (acidic, e.g., HNO_3_, HCl)	pH < 1 or >5; milder catalysts (e.g., citric acid for borates)	optimal TEOS hydrolysis and Si-OH formation; rapid, uncontrolled condensation/poor mesostructure at high pH; slower hydrolysis/better ordering with milder catalysts	[14,16,21,31,36,80]
Aging temperature	60–80 °C	room temperature (20–25 °C); >100 °C	accelerates EISA and micelle ordering (24–48 h); slow EISA/extended aging (48–72 h) at RT; rapid solvent loss/disordered structures at high temperature	[16,80]
Aging time	24–48 h (most P123 syntheses); 12–24 h (borate glasses)	<12 h; >72 h	insufficient consolidation/weak structures; minimal additional benefit/increased synthesis time	[16,36,80]
Calcination temperature	600–700 °C (silicate MBGs); 500–600 °C (borate/phosphate)	<500 °C; >700 °C (silicates); 800 °C (Ce-MBGs)	incomplete template removal/residual organics; mesopore collapse/partial crystallization (e.g., wollastonite, CaSiO_3_)/significant surface area loss	[59,81]
Calcination heating rate	slow (0.5–1 °C/min)	fast (>5 °C/min)	gradual template decomposition/preserves ordering; rapid gas evolution/damages mesostructure	[59,80,81]
Calcination holding time	3–6 h	shorter times; longer times	carbon residues may remain; sintering/pore shrinkage may occur	[14,16]

**Table 2:** Mechanistic comparison of SDAs in calcium-containing BMG systems.

Parameter	P123/F127 (Non-Ionic)	CTAB (Cationic)
Primary surfactant–Ca^2+^ interaction	coordination of Ca^2+^ with ether oxygen atoms in PEO chains	electrostatic screening and competition between Ca^2+^ and CTA^+^ for interaction with silicate species
Micelle stabilization mechanism	steric stabilization provided by hydrated PEO corona combined with Ca^2+^ coordination	electrostatic repulsion between positively charged headgroups
Effect of increasing Ca^2+^ concentration	generally stabilizing up to moderate–high CaO contents	strongly destabilizing at moderate CaO contents
Dominant disruption mechanism at high Ca^2+^	changes in solvation environment and formation of Ca-rich phases	charge screening, increased ionic strength, and micelle aggregation
Sensitivity to ionic strength	low to moderate	high
Typical mesostructural outcome	ordered mesopores retained over a wider CaO compositional range	early loss of long-range mesostructural order
Relevance for MBG design	suitable for high-bioactivity compositions requiring elevated CaO contents	primarily applicable to low–moderate CaO compositions

**Table 3:** Comparative features of common SDAs used to generate MBGs.

Feature	CTAB (Cationic Surfactant)	Pluronic P123 (Nonionic TBC)	Pluronic F127 (Nonionic TBC)	Refs.
Mechanism	electrostatic interactions with anionic silica species	H-bonding and van der Waals with PEO blocks	H-bonding and van der Waals with PEO blocks	[14]
Molecular weight	~364.45 Da	~5800 Da	~12,600 Da	[14,16]
Micelle size	small (~2–3 nm)	medium (~5–7 nm)	large (~8–10 nm)	[14]
Typical pore size	2–4 nm	4–7 nm (often ~4.2 nm)	5–8 nm (often ~5.8 nm)	[14,16,83]
Pore ordering	hexagonal (p6 mm)	hexagonal or wormlike	hexagonal or cubic	[14]
Surface area	very high (400–500 m^2^/g)	high (300–400 m^2^/g)	moderate (200–300 m^2^/g)	[14]
Pore volume	low (0.2–0.4 cm^3^/g)	high (0.4–0.7 cm^3^/g)	high (0.5–0.8 cm^3^/g)	[14]
Wall thickness	thin (~2–3 nm)	medium (~3–5 nm)	thick (~5–7 nm)	[14]
Sensitivity to Ca^2+^	high (disrupts mesophase ordering)	moderate (less sensitive)	moderate (less sensitive)	[14]
Drug loading capacity	limited (for small pores)	high	very high (for large molecules)	[14]
Primary advantages	very high surface area, uniform small pores	good balance of SA/PV, less Ca^2+^ sensitive	large pores, thick walls, good for protein delivery	-
Primary limitations	high Ca^2+^ sensitivity, lower pore volume	smaller pores than F127, less structural stability at high temperatures	lower surface area than CTAB/P123	-

**Table 4:** Summary of therapeutic ions doped in MBGs and their key biological effects.

Therapeutic Ion	Doping Range (mol%)	Key Biological Effects	Example of Application	Refs.
Strontium (Sr^2+^)	0–10 mol% SrO	enhances osteogenesis, inhibits osteoclast activity, promotes HA formation	bone regeneration in sheep model	[5,80]
Copper (Cu^2+^)	1–5 mol% CuO	antibacterial, angiogenic (stimulates VEGF), osteogenic	bone tissue engineering, dental composites	[12,28,77,86]
Zinc (Zn^2+^)	1–5 mol% ZnO	antibacterial, osteogenic, promotes bone metabolism, wound healing	bone regeneration, wound healing	[21,78,98,100]
Boron (B^3+^)	0–5 mol% B_2_O_3_	osteogenic, angiogenic, increases network connectivity, improves mechanical properties	enhanced bioactivity, muscle tissue application	[59,99]
Cerium (Ce^3+^/Ce^4+^)	1–5 mol% CeO_2_	antioxidant, anti-inflammatory (ROS scavenging), osteogenic	bone regeneration, caries management	[79,81,101,103]
Cobalt (Co^2+^)	1–3 mol% CoO	angiogenic	tissue engineering	[20,31]
Gallium (Ga^3+^)	1–3 mol% Ga_2_O_3_	antibacterial, immunomodulatory	immunomodulatory PEEK implants	[108,109]
Manganese (Mn^2+^)	1–3 mol% MnO	osteogenic	osteogenic differentiation	[25,73]
Iron (Fe^3+^)	5–10 mol% Fe_2_O_3_	magnetic properties (for hyperthermia), drug release	cancer therapy (melanoma)	[57]
Magnesium (Mg^2+^)	Up to 10 mol% MgO	osteogenic, biodegradable, cardioprotective	myocardial ischemia/reperfusion	[3,110]

**Table 5:** Comprehensive overview of characterization techniques for MBGs.

Technique	Purpose	Key Information Obtained	Advantages	Limitations	Refs.
SAXS	confirms mesoscopic ordering, lattice parameters	degree of ordering (hexagonal, wormlike), pore periodicity	non-destructive, confirms mesostructural ordering	lower resolution than TEM, limited to bulk structure	[59]
TEM	direct visualization of mesopore structure and ordering	pore channel morphology, wall thickness, ordering quality, particle morphology/size	direct visualization, confirms templating success, amorphous nature	complex/thin sample prep, beam damage, small sampling area, expensive	[13,16]
BET/BJH	quantifies surface area, pore volume, pore size distribution	specific surface area, total pore volume, pore size distribution, isotherm type	quantitative data, sensitive to structural changes	requires dry samples, theoretical models, does not distinguish surface chemistry	[13,14,16]
SEM	examines surface morphology, particle/scaffold architecture	particle shape/size, surface roughness/porosity, aggregation, macroporosity, scaffold interfaces, HA formation	direct surface imaging, good for larger features, elemental mapping (with EDX)	lower resolution than TEM, conductive coating needed, surface charging, limited depth info	[1,78]
XRD	confirms/detects amorphousness/crystallization/phase changes	amorphous vs. crystalline phases, mesopore ordering (low angle), HA formation	reliable confirmation of glassy nature, non-destructive, bulk analysis	no local atomic info, minor inclusions escape detection, needs standards	[10,81]
FTIR	identifies functional groups, network structure, bonding environment	silicate network formation, organic residues, C-H bands (surfactant removal), HA formation	non-destructive, fast, identifies chemical bonds, sensitive to structural changes	qualitative not quantitative, limited spatial resolution, overlapping peaks	[14,21]
NMR	provides detailed info on network structure and connectivity	Q^n^ species (^29^Si), BO_3_/BO_4_ units (^11^B), phosphate environment (^31^P)	detailed local atomic arrangement, network connectivity	specialized equipment, requires specific nuclei, complex data interpretation	[59]
EDS/EDX	elemental composition and distribution	stoichiometry, dopant distribution, homogeneity	elemental mapping, semi-quantitative, coupled with SEM/TEM	limited accuracy, sample matrix effects, destructive (if standalone digestion)	[13]
SBF Immersion	evaluates in vitro bioactivity	HA layer formation (XRD/SEM), “cauliflower” morphology, Ca/P ratio	direct assessment of bioactivity, standardized protocol	in vitro may not fully reflect in vivo, qualitative assessment can be subjective	[80]
Ion release studies	quantifies release of therapeutic ions	release kinetics (burst, sustained, plateau phases), ion concentration	quantitative, essential for drug delivery/therapy	requires sensitive techniques (ICP-OES/MS), complex if multiple ions/matrices, destructive (sample dissolution)	[14]
Cell viability/proliferation	assesses cytotoxicity and cell growth	metabolic activity, cell count, DNA quantification, live/dead assay	direct assessment of biocompatibility	in vitro model, may not predict in vivo response, potential for assay interference	[16]
Osteogenic differentiation	evaluates bone cell maturation	ALP activity, OCN/BSP expression, mineralization (Alizarin Red S)	osteoinductivity direct assessment	in vitro model, complex assays, often long duration	[80,85,100]
Antibacterial activity	measures antimicrobial efficacy	zone of inhibition, CFU count, live/dead staining	direct assessment of antimicrobial properties	in vitro model, specific bacterial strains, concentration dependence	[1,86]
In vivo studies	evaluates performance in living systems	bone volume/density (µCT), histology, angiogenesis, immune response, mechanical strength	direct assessment of physiological relevance	high cost, ethical considerations, complex logistics, species-specific results	[5,98]

**Table 7:** Summary of future research directions and potential impact.

Research Area	Specific Direction	Potential Impact/Benefits	Enabling Technologies	Refs.
Advanced synthesis methods	eco-Friendly Synthesis (bio-based SDAs, plant extracts, reduced solvents)	reduced environmental footprint, safer manufacturing, sustainable biomaterials	green chemistry, biotechnology	[111]
	precision manufacturing (aerosol-assisted, microfluidics, multi-material 3D printing)	customized geometries, improved homogeneity, scalable production	advanced additive manufacturing, microfluidic devices, robotics	[1,29,56]
Multifunctional MBGs and smart materials	stimuli-Responsive MBGs (pH, T, enzyme, magnetic, light, ultrasound)	on-demand drug release, targeted therapy, adaptive implants	advanced polymer chemistry, sensor integration, remote activation	[115]
	multifunctional platforms (theranostic, combined therapeutic + diagnostic)	integrated diagnosis and therapy, personalized treatment, real-time monitoring	nanotechnology, advanced imaging, biosensors	[115]
Advanced characterization and modeling	in situ characterization (e.g., synchrotron SAXS)	real-time process understanding, optimized synthesis parameters	synchrotron radiation, advanced spectroscopic techniques	[59]
	computational modeling (MD simulations, predictive models)	accelerated material design, prediction of properties, reduced experimental burden	high-performance computing, AI/machine learning	[14]
Personalized medicine and expanding applications	patient-specific designs (3D printing, tailored ion doping)	customized implants, optimized therapeutic effect for individual patients	medical imaging (CT/MRI), AI algorithms, advanced 3D printing	[1,29,56]
	integration with stem cells (MBG-stem cell composites)	enhanced regenerative capacity, complex tissue reconstruction	cell biology, tissue engineering protocols	[29,56]
	novel therapeutic areas (neural, cardiac tissue engineering, biosensors)	solutions for unmet medical needs (e.g., nerve regeneration, heart repair)	neurobiology, electrophysiology, enzyme immobilization	[47,50,57,74,110]
Advanced composites	hybrid organic-inorganic materials (with 2D materials like graphene, MXenes)	enhanced mechanical strength, electrical conductivity, tunable degradation, multi-functionality	advanced material science, nanotechnology, interfacial engineering	[29,30]

## Data Availability

No new data were created or analyzed in this study. Data sharing is not applicable to this article.

## Abbreviations

BET	Brunauer–Emmett–Teller
BMSCs	bone marrow mesenchymal stem cells
CaO	calcium oxide
ccCs	catechol-conjugated chitosan
CT	computed tomography
CTAB	hexadecyltrimethylammonium bromide
DLS	dynamic light scattering
EDTA	ethylenediaminetetraacetic acid
EDX	energy dispersive X-ray Spectroscopy
EISA	evaporation-induced self-assembly
EO	ethylene oxide
FTIR	Fourier transform infrared spectroscopy
HA	hydroxyapatite
HCA	hydroxyl carbonate apatite
HRTEM	high-resolution transmission electron microscopy
ICP-OES	Inductively Coupled Plasma Optical Emission Spectroscopy
LLA	polylactic acid
MRI	magnetic resonance imaging
NAC	N-acetylcysteine
NMR	nuclear magnetic resonance
NPs	nanoparticles
P4VP	poly(4-vinylpyridine)
PCL	polycaprolactone
PEG	polyethylene glycol
PHB	Cs-poly(3-hydroxybutyrate)-chitosan
Pluronics P123, F127, and F68	poly(ethylene glycol)-*block*-poly(propylene glycol)-*block*-poly(ethylene glycol)
PMMA	poly(methyl methacrylate)
PPO	poly(propylene oxide)
PU	polyurethane
PVA	polyvinyl alcohol
PVPy	polyvinylpyrrolidone
ROS	reactive oxygen species
RT	room temperature
SAED	selected area electron diffraction
SAXS	small-angle X-ray scattering
SBF	simulated body fluid
SEM	scanning electron microscopy
TBC	triblock copolymer
TEM	transmission electron microscopy
TEOS	tetraethyl orthosilicate
TEP	triethyl phosphate
VEGF	vascular endothelial growth factor
XRD	X-ray diffraction
